# Reflectance Confocal Microscopy Can Help Differentiate Adult Xanthogranulomatous Disease from Xanthelasma—A Case Report

**DOI:** 10.3390/jcm14041359

**Published:** 2025-02-18

**Authors:** Larysa Krajewska-Węglewicz, Monika Dźwigała, Piotr Sobolewski, Anna Wasążnik-Jędras, Irena Walecka

**Affiliations:** 1 Department of Ophthalmology, National Institute of Medicine of the Ministry of the Interior and Administration, 02-507 Warsaw, Poland; 2 Department of Dermatology, National Institute of Medicine of the Ministry of the Interior and Administration, 02-507 Warsaw, Poland; 3 Department of Pathomorphology, National Institute of Medicine of the Ministry of the Interior and Administration, 02-507 Warsaw, Poland

**Keywords:** xanthogranuloma, xanthoma, reflectance confocal microscopy

## Abstract

**Background**: Adult xanthogranulomatous disease (AXD) is a rare histiocytic disorder with systemic potential, while xanthelasma palpebrarum (XP) is a common xanthoma often linked to lipid disorders. Differentiating these conditions is challenging due to overlapping features. Reflectance confocal microscopy (RCM), a non-invasive imaging tool, offers high-resolution visualization of skin structures and may aid diagnosis. **Methods**: We present a 71-year-old woman with periocular lesions. RCM was used to evaluate the lesions, identifying cellular and structural features. The findings were confirmed through histopathology, followed by surgical excision. Postoperative monitoring utilized RCM and LC-OCT. **Results**: RCM identified Touton giant cells, foamy histiocytes, and fibrosis, helping to distinguish xanthogranuloma from xanthelasma. Histopathology confirmed the diagnosis, and the patient underwent successful lesion excision without complications. **Conclusions**: This case underscores RCM’s utility as a diagnostic adjunct for differentiating AXD from XP in sensitive regions like the periocular area. The combined use of RCM and LC-OCT enhances monitoring for recurrence. While histopathology remains the diagnostic gold standard, RCM shows promise as a non-invasive tool, warranting further research to validate its role and develop standardized clinical protocols.

## 1. Introduction

Xanthogranuloma (XG) is a benign, rare form of non-Langerhans histiocytosis characterized by the proliferation of histiocytes and lipid-laden macrophages. It can manifest in various forms, primarily in two demographic groups: children, as juvenile xanthogranuloma (JXG), and adults, as part of adult xanthogranulomatous disease (AXD). While JXG is typically confined to the skin and often resolves spontaneously, adult-onset XG may involve extracutaneous sites, such as the periocular region, respiratory tract, and other internal organs, with the potential for systemic implications [1]. Histologically, XG lesions exhibit dense dermal infiltration of histiocytes, often with Touton giant cells. These giant cells contain a central area of lipid-laden cytoplasm surrounded by a ring of nuclei. Other features include foamy histiocytes, lymphocytes, plasma cells, and occasional eosinophils. Fibrosis and deeper infiltration are more common in adult-onset XG compared to its juvenile counterpart, contributing to its distinct clinical course [2].

Epidemiologically, JXG predominantly affects infants and young children with no gender predilection, whereas adult-onset XG is exceedingly rare, with a chronic and sometimes systemic course. Variants such as necrobiotic xanthogranuloma and Erdheim–Chester disease are often associated with significant morbidity due to systemic involvement [3,4].

In contrast, xanthelasma palpebrarum (XP) is the most common form of cutaneous xanthoma. It presents as soft, yellowish plaques located primarily on the medial aspects of the upper and lower eyelids. Although XP is largely considered a cosmetic concern, it can occasionally indicate underlying systemic lipid disorders such as hypercholesterolemia or familial dyslipidemia. Histopathologically, XP lesions consist of foam cells or xanthoma cells, which are histiocytes loaded with intracellular lipid droplets. These are predominantly esterified cholesterol, located within intracytoplasmic vacuoles in the upper reticular dermis, often around blood vessels and adnexal structures [5]. Unlike XG, XP lacks a significant inflammatory component, fibrosis, or deeper tissue involvement [6].

XP typically affects middle-aged to older adults, with a higher prevalence in women. Studies have shown that approximately 50% of individuals with XP have an associated lipid metabolism disorder, making its identification clinically significant as a marker for systemic dyslipidemia and cardiovascular risk [7].

Distinguishing between XG and XP is crucial due to differences in clinical presentation, histology, and potential systemic involvement. XG lesions are often nodular or plaque-like, with deeper tissue extension and an associated inflammatory infiltrate, including Touton giant cells and fibrosis. These features are absent in XP, which presents as flat, yellowish plaques limited to the superficial dermis. Furthermore, while XP is relatively common, particularly in individuals with lipid disorders, XG remains rare, with a broader range of potential systemic implications [8].

Despite these histopathological and clinical distinctions, differentiation can be challenging, particularly in cases of rare adult-onset XG. Misdiagnoses have been reported, such as in the case described by Dosari et al., where a patient with AXD was initially misdiagnosed with XP due to a lack of clinical suspicion [9]. The rarity of XG, combined with its overlap in appearance with other xanthomatous lesions, underscores the importance of heightened awareness among clinicians.

In recent years, non-invasive diagnostic techniques have gained prominence in the evaluation of dermatological conditions, including xanthomatous lesions. Reflectance confocal microscopy (RCM) offers real-time, in vivo imaging of the skin at cellular resolution. RCM uses a near-infrared power laser which emits monochromatic coherent light to scan a focal point in the skin [10,11]. The subsequent reflected light from the tissue, passing through a gating pinhole, arrives at the detector, which reconstructs a grayscale image based on the relative refractive indices of the tissue elements. Melanin and keratin, which have the highest refractive indices, appear brighter than surrounding tissue elements [12]. The basic RCM image displays a horizontal (en face) field of view of 0.5 mm × 0.5 mm, but larger mosaic images up to an area of 8 mm × 8 mm (Vivablock) can be obtained. The clinician can control probe movement in the horizontal and vertical plane up to a depth of 250–300 µm.

While its utility in the differential diagnosis of xanthomas has not been extensively studied, its application could provide a valuable, non-invasive alternative to biopsies, particularly in delicate areas such as the periocular region, where surgical interventions may compromise function or aesthetics [13].

## 2. Case Presentation

**Figure 1:**
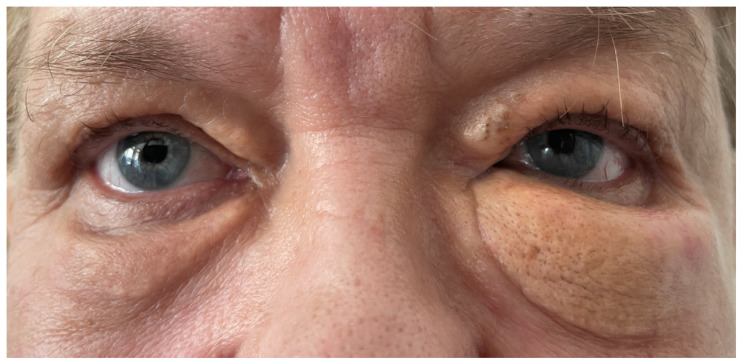
XP of the inner canthus of both upper eyelids. XG of the medial portion of the left upper eyelid and both lower eyelids.

A 71-year-old woman presented with a three-year history of progressively enlarging periocular lesions (Figure 1). The patient presented with yellowish, firm, immobile nodules on the upper and lower eyelids. Smaller, soft plaques localized to the inner canthus were also observed. Diagnostic investigations included clinical examination, magnetic resonance imaging (MRI), RCM, and histopathological biopsy. MRI ruled out intraorbital extension. RCM imaging was performed using a high-resolution, handheld confocal microscope (Vivascope 3000, MAVIG GmbH, Munich, Germany) to assess cellular and tissue-level details.

**Figure 2:**
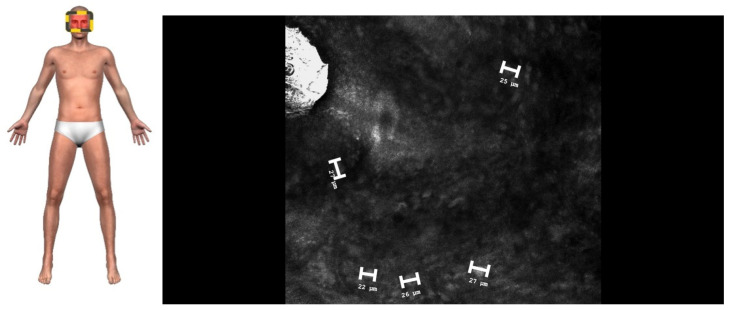
A confocal image of XP showing hyper-reflective cells in the upper dermis, corresponding to foamy histiocytes—marked with a size marker (0.5 × 0.5 mm, 30× magnification).

**Figure 3:**
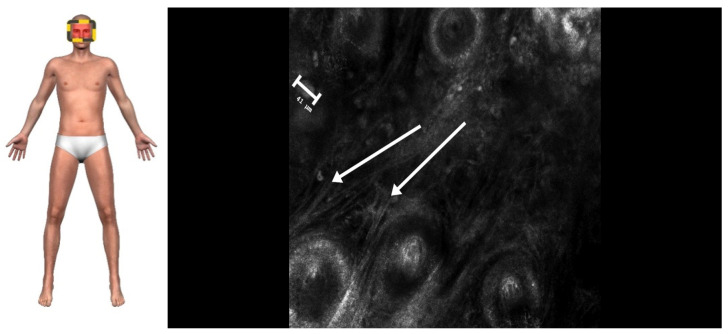
A confocal image of XG, indicating parallel collagen fibers in the dermis (arrows) corresponding to fibrosis in histology. The large, multinucleated hyper-reflective cells (size marker) correspond to Touton giant cells (0.5 × 0.5 mm, 30× magnification).

RCM imaging of the plaques revealed medium-to-high refractive foam-like cells in the superficial and middle layers of the dermis that corresponded to the lipid-laden foamy histiocytes in histopathology, characteristic of XP. The cells were approximately 22–33 μm in size (Figure 2). Larger nodules exhibited a honeycomb pattern; high refractive horseshoe-like, disc-shaped, ring-like cells; fibrosis; and deeper dermal involvement, consistent with XG. The giant cells measured approximately 40–80 μm (Figure 3).

**Figure 4:**
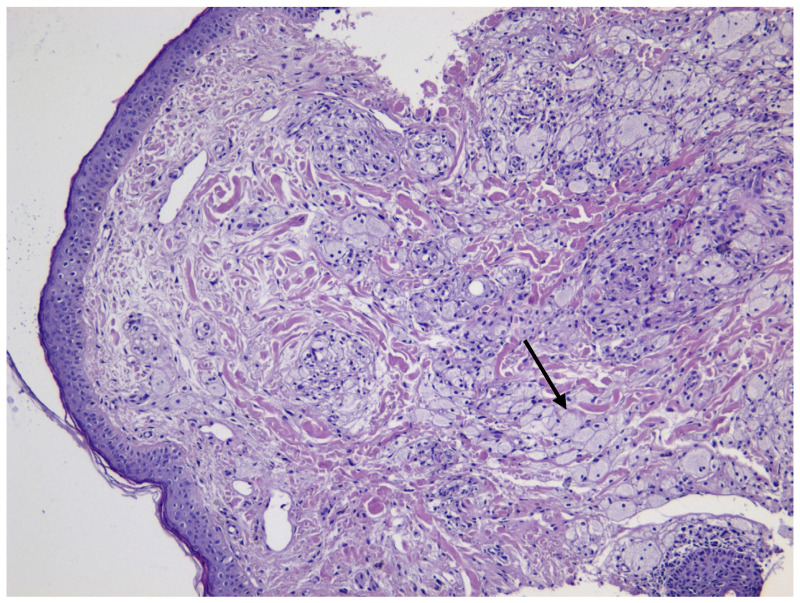
XP histology: arrow indicates small clusters of foamy histiocytes in the dermis (H&E, 10× magnification).

**Figure 5:**
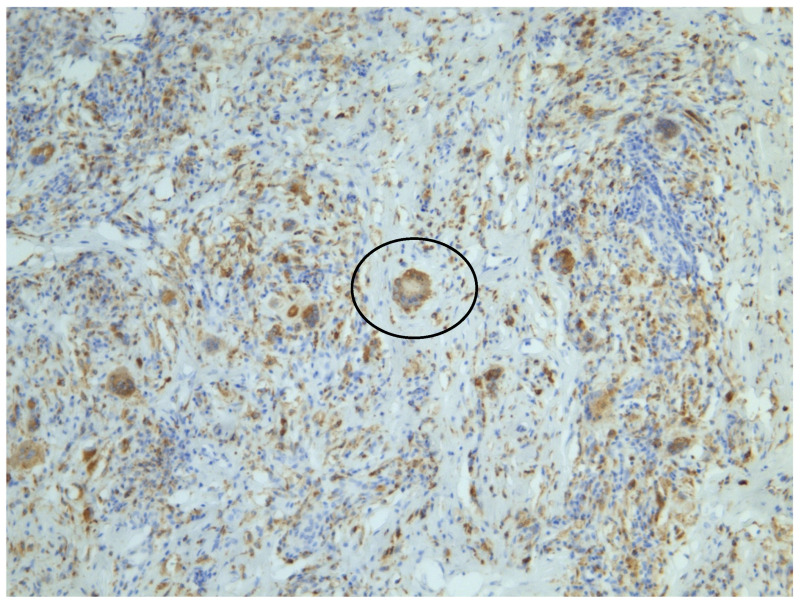
XG histology: Touton giant cell highlighted (CD68 immunohistochemical staining, 10× magnification).

Histological findings confirmed the diagnosis, with foamy histiocytes measuring approximately 30 µm in XP (Figure 4) and Touton giant cells ranging from 40 to 86 µm in XG (Figure 5). Surgical excision of the lesions with flap reconstruction was performed without complications, and systemic evaluation showed no evidence of extracutaneous involvement. Follow up was conducted with the use of LC-OCT.

## 3. Discussion

This case highlights the utility of reflectance confocal microscopy (RCM) as a non-invasive diagnostic tool in differentiating XG from XP in the periocular region. While both conditions present with yellowish lesions, their clinical implications, histopathology, and systemic associations differ significantly, necessitating an accurate diagnosis for appropriate management.

### 3.1. Histopathological Distinction

XG, particularly AXD, is characterized histologically by dermal infiltration of foamy histiocytes, Touton giant cells, chronic inflammatory infiltrates, and varying degrees of fibrosis. Touton giant cells, a hallmark of XG, are multinucleated histiocytes with lipid-laden cytoplasm and a peripheral ring of nuclei, reflecting disordered lipid metabolism within macrophages [2,14]. In contrast, XP lesions consist primarily of foam cells—lipid-laden histiocytes—localized to the superficial dermis, with minimal inflammatory infiltrates and absent fibrosis [8]. These distinctions were corroborated in this case through histopathological findings.

The use of RCM provided real-time in vivo imaging of these histological features, demonstrating foamy histiocytes, Touton giant cells, and fibrosis, which aligned closely with biopsy results. Previous studies have emphasized RCM’s ability to visualize key cellular structures, offering a promising diagnostic alternative to histopathology [15].

### 3.2. Advantages of RCM in Periocular Diagnosis

The periocular region presents unique challenges for diagnosis and treatment due to its functional and aesthetic significance. Traditional biopsies in this region carry risks of scarring, functional impairment, and patient discomfort. RCM has emerged as a valuable, non-invasive imaging modality capable of providing cellular-resolution images of superficial and mid-dermal layers [16]. In our case, RCM successfully distinguished features in XG, such as Touton giant cells and fibrosis, which are absent in XP.

### 3.3. RCM as an Emerging Diagnostic Tool

Several prior studies have underscored the utility of RCM in diagnosing a range of dermatological conditions, including melanomas, basal cell carcinoma, and inflammatory dermatoses [17]. However, its role in histiocytic disorders such as XG and XP remains underexplored. Haroon et al. [18] and Lovato et al. [19] described RCM findings in cases of XG, where the imaging revealed cellular features consistent with histopathology. Similarly, Lacarrubba et al. [20] highlighted RCM’s utility in evaluating XGs, reporting its ability to differentiate Touton giant cells that appeared as roundish giant cells surrounded by a highly refractive peripheral ring due to the cytoplasm rich in lipids. The findings of Sun et al., who compared benign XG with XL using RCM, align with our results, confirming that the features of XG and XL are distinctive when evaluated with RCM [21]. This case series supports our findings, highlighting that RCM is effective in distinguishing XG from XL. However, our paper presents a unique case of AXD occupying the periocular region. Notably, the XG lesion is adjacent to an XL lesion, offering a rare and valuable opportunity to explore histopathological differences between the two conditions within the same patient.

RCM offers several advantages. It reduces the need for surgical biopsies, particularly in cosmetically sensitive areas like the periocular region. It also eliminates the need for local anesthesia and sutures, making it well tolerated by patients. Additionally, unlike traditional biopsy, RCM allows the entire lesion to be evaluated in vivo.

### 3.4. Clinical Implications

While XP is often associated with underlying lipid metabolism disorders, such as hypercholesterolemia and familial dyslipidemia, XG—particularly AXD—may signal systemic involvement, including organ infiltration and hematologic abnormalities [1]. Distinguishing between these entities is critical, as XP management focuses on lipid profile optimization and cosmetic interventions, while AXD requires systemic evaluation and surveillance.

In our case, systemic evaluation ruled out extracutaneous involvement, consistent with localized periocular XG. Nonetheless, cases of XG with systemic manifestations, such as necrobiotic xanthogranuloma and Erdheim–Chester disease, have been reported and often require multidisciplinary management [3,4]. These variants pose significant morbidity and mortality, highlighting the importance of accurate diagnosis.

### 3.5. Future Directions

The findings from this case contribute to the growing body of evidence supporting RCM as a valuable adjunct in dermatologic diagnosis. Future research involving larger cohorts is needed to validate the diagnostic accuracy and reliability of RCM in differentiating histiocytic disorders. Comparative studies between RCM and traditional histopathology could further clarify its role in clinical practice. Additionally, integrating RCM with emerging technologies, such as LC-OCT and artificial intelligence-based image analysis, may enhance diagnostic precision and facilitate broader clinical adoption.

### 3.6. Comparison of RCM with Other Modalities

RCM, dermatoscopy, LC-OCT, and high-resolution ultrasound (HRUS) each offer unique advantages and limitations in the evaluation of skin lesions such as XG and XL.

#### 3.6.1. Dermatoscopy

Dermatoscopy is a widely used non-invasive imaging technique that allows for detailed en face visualization of the skin, extending down to the mid-dermis. It is an essential tool in dermatologic diagnostics, particularly effective for evaluating conditions that involve the epidermis and the superficial dermis. This method provides insights into pigment distribution, vascular structures, and superficial lesions. However, its diagnostic sensitivity and specificity are highly dependent on the clinician’s expertise and experience, as it requires skill to distinguish subtle features. Unlike more advanced techniques like reflectance confocal microscopy (RCM), dermatoscopy lacks cellular resolution. As a result, it cannot visualize fine morphological details, such as histiocytic infiltrates, Touton giant cells, or other intracellular changes, that could be critical for precise diagnosis.

#### 3.6.2. LC-OCT (Line-Scan Optical Coherence Tomography)

**Figure 6:**
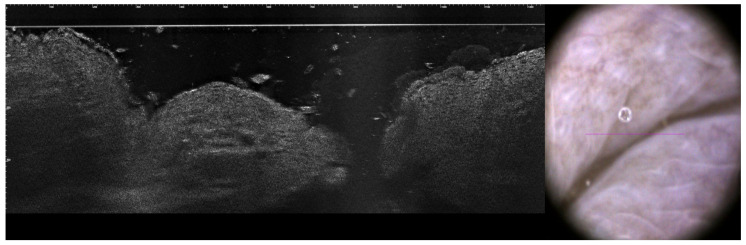
Vertical OCT tomogram and dermoscopy image six months after surgical excision with flap reconstruction. Normal skin architecture with no signs of XG recurrence. Scale bar indicating 100 μm.

LC-OCT provides high-resolution en face and cross-sectional imaging, enabling a penetration depth of up to 2 mm. This depth allows LC-OCT to evaluate the overall architecture of lesions and their boundaries, making it particularly useful for assessing lesion depth and aiding in pre-surgical planning. However, LC-OCT does not achieve cellular resolution, unlike RCM, and therefore cannot visualize detailed histological structures such as Touton giant cells or foam cells [22]. In the context of XG and XL, LC-OCT offers valuable complementary information to RCM by delineating lesion thickness and subdermal involvement. The combined use of RCM and LC-OCT enhances the understanding of the morphological architecture of nodular lesions, aiding in the differentiation between benign and malignant entities. While RCM and LC-OCT individually provide valuable information in the evaluation of skin lesions, their combined use offers a more comprehensive assessment. This multimodal approach enhances diagnostic precision, particularly in distinguishing between benign and malignant lesions, and holds promise for improving patient management strategies. Further studies are warranted to elucidate the specific imaging features of XG using these modalities. In the presented case, we used both RCM and LC-OCT to monitor sub-flap recurrence following surgery (Figure 6). After the excision of the lesion, especially when a flap or skin transplant reconstruction is employed, monitoring recurrence in deeper structures can be challenging. LC-OCT provides valuable insights in this context.

#### 3.6.3. High-Frequency Ultrasound (HFUS)

Ultrasound is a non-invasive imaging technique based on the measurement of sound wave reflections from the tissues of the body. While lower frequencies permit the exploration of deeper structures such as the internal organs, high-frequency ultrasound (HFUS), with transducer frequencies of 20 MHz or more, has a lower depth of tissue penetration but produces a higher-resolution image of tissues and structures closer to the skin’s surface [23]. Indeed, frequencies of 20 MHz to 25 MHz allow for the visualization of skin up to the dermis, while higher frequencies of 50 MHz and above visualize only the epidermis [24]. While HFUS is effective for assessing tumor depth and overall lesion structure, its resolution is lower than that of RCM. For XG, HFUS can help to visualize deep fibrotic stroma and diffuse dermal infiltration, but it lacks the capability to identify cellular features such as Touton giant cells. HFUS is also highly operator-dependent, requiring significant training for accurate interpretation.

RCM offers near-histological resolution, providing detailed en face images of the superficial dermis and epidermis. It is uniquely capable of identifying cellular-level features, such as foam cells and Touton giant cells, which are critical for distinguishing XG from XL. However, its limited penetration depth restricts its ability to evaluate deeper dermal structures, making it useful to combine RCM with techniques such as LC-OCT or HFUS for comprehensive lesion assessment.

### 3.7. Limitations

The limitations are mostly due to the inherent nature of this technology, such as en face visualization of the tissue, limited depth of imaging, small field of view (FOV) images (when using the handheld RCM device), grayscale images, and a lack of cellular specificity (i.e., the inability to differentiate dendritic melanocytes from Langerhans cells). Largely due to these difficulties with image interpretation and high cost, the adoption of RCM remains limited worldwide, despite its usefulness [10]. The cost-effectiveness of RCM remains a significant limitation. The devices are expensive to acquire and maintain [25], and the need for extensive operator training [26] adds to the overall cost of implementation. For these reasons, RCM is mainly available in academic centers. Furthermore, the limited penetration depth of 250–300 microns restricts the visualization of deeper dermal structures, where critical pathological features such as diffuse infiltration or fibrosis may reside [27]. These factors make RCM less accessible in some clinical settings, particularly in resource-limited regions.

Deeper structures produce weak reflectance signals due to the scattering and absorption of light by overlying tissues, leading to poor image quality and resolution at greater depths [28]. XG often exhibits diffuse infiltration and fibrosis, which extend beyond the papillary dermis. However, Touton giant cells reside in the superficial to mid-dermis, as Sun et al. showed in their study [21]. Without the visualization of this fibrosis, the full extent of the lesion cannot be assessed, which may hinder accurate classification or staging. To overcome these limitations, RCM is best used in conjunction with deeper-penetrating techniques, such as dermoscopy, LC-OCT, HFUS, or histopathology. Biopsy remains essential for confirming features located in the deeper dermis and establishing a definitive diagnosis.

RCM is particularly useful for monitoring lesions over time. For instance, XG, in its early stages, may show changes in the papillary dermis that RCM can capture, allowing clinicians to track progression or resolution.

## 4. Conclusions

This case highlights RCM’s potential as a complementary, non-invasive diagnostic tool for distinguishing XG from XL, particularly in the periocular region where biopsy risks are significant. While RCM cannot replace histopathology as the gold standard, it can aid in lesion evaluation and patient management. The combined use of RCM and LC-OCT enhances monitoring for recurrence. Further studies on larger cohorts of patients are warranted to establish standardized protocols and validate this approach’s broader clinical utility.

## Data Availability

Data are contained within the article.
